# Patient satisfaction and perioperative data after breast surgery in tumescent local anaesthesia

**DOI:** 10.1007/s00404-026-08524-x

**Published:** 2026-07-18

**Authors:** M. Meffert, B. Schoenfisch, S. Guergan, I. Gruber, U. Hoopmann, G. Helms, C. Roehm, J. Pasternak, C. Haupt, E. Oberlechner, S. Y. Brucker, M. Hahn, B. Boeer

**Affiliations:** Department of Women’s Health, University Hospital of Tuebingen, Calwerstraße 7, 72076 Tuebingen, Germany

**Keywords:** Breast surgery, Tumescent local anaesthesia, Patient satisfaction, Tumescent volume

## Abstract

**Purpose:**

Tumescent local anaesthesia (TLA) is an upcoming alternative to general anaesthesia. No data exists regarding patient satisfaction, necessary volume of TLA, frequency of supplementary analgosedation, and associated complication rate after different kinds of breast surgeries in TLA.

**Methods:**

In this retrospective study 104 patients, who underwent breast surgery in TLA between 1/2022 and 1/2023, answered a questionnaire. Patients were categorized according to histology and surgical complexity into two groups: those with benign histology undergoing minor procedures (outpatients) and those with malignant histology undergoing more complex surgical procedures (inpatients). Perioperative data was retrieved from the patients’ files.

**Results:**

96.1% of all patients were (very) satisfied with the surgery, with inpatients being significantly more satisfied (p = 0.022). No significant differences were observed between the groups regarding perception of TLA-infiltration, likelihood of recommending the procedure in TLA, or willingness to undergo the procedure again in TLA. Six patients (5.8%) had postoperative complications with no significant difference in frequency of complications. Outpatients needed significantly less TLA-volume (184.7ml ± 131.4 ml), compared to inpatients with a total TLA-volume of 473.3 ml ± 205.2 ml (p < 0.001). 25 patients (24.0%) opted for an intraoperative midazolam sedation (mean 1.5 mg, SD 0.9mg), predominantly in more extensive procedures.

**Conclusion:**

Patients’ high satisfaction and recommendation level and the low rate of complications are convincing reasons to offer TLA as an alternative to general anaesthesia for a large spectrum of breast surgeries.

**Supplementary Information:**

The online version contains supplementary material available at 10.1007/s00404-026-08524-x.

## What does this study add to the clinical work?

**Table:** 

This study provides data regarding patient satisfaction, the volume of tumescent local anaesthesia required to achieve satisfactory anaesthesia and the associated complication rate after different types of breast surgery in tumescent local anaesthesia.	

## Introduction

Breast surgery is predominantly performed under general anaesthesia (GA) [1]. However, elderly patients frequently present with significant comorbidities, making GA a relevant source of perioperative morbidity [2, 3]. In recent decades regional analgesic techniques have been introduced like locoregional blocks, but they are almost exclusively applied as adjuncts to GA rather than as true alternatives [4, 5]. Carlson et al. reported successful mastectomy in four ASA IV patients (mean age 72 years) using tumescent local anaesthesia (TLA) [6]. TLA is an established and safe technique for head and neck skin cancer surgery and lymph node dissection [7, 8]. In 2023 TLA was described as a sole anaesthetic alternative to GA in breast surgery [1], however, data on patient satisfaction and perioperative data related to TLA surgery of the breast have not yet been published.

This study primarily assessed patient satisfaction after breast surgery in TLA. Secondary endpoints included tumescent volume relative to procedure type, need for supplementary analgosedation and complication rates.

## Material and methods

### Study design

**Fig. 1:**
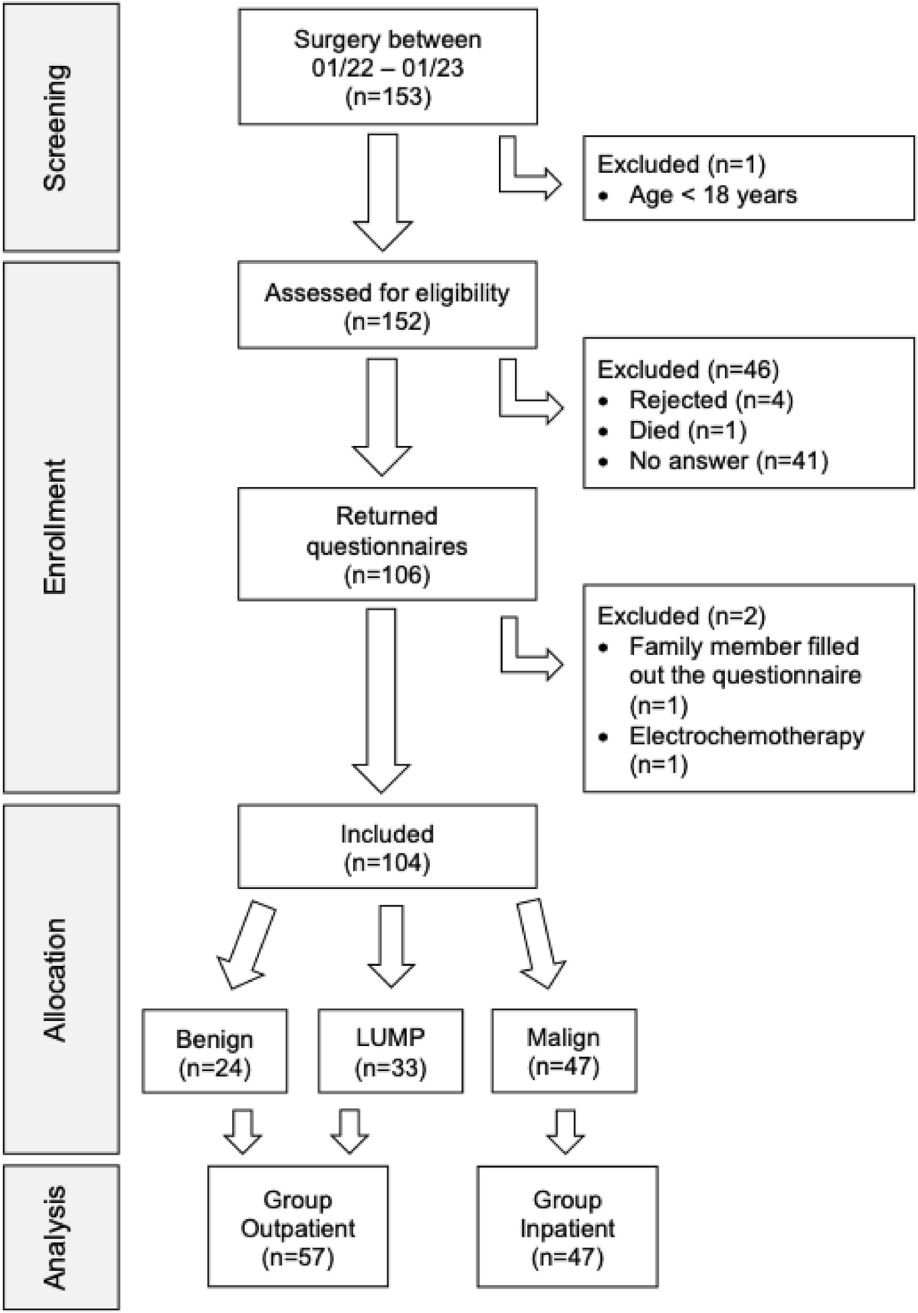
Flow-chart: Response rate of the study and included patients

Between January 2022 and January 2023, 153 patients who underwent breast surgery in TLA at the Department of Women’s Health, University of Tuebingen (Germany) were screened. Of these, 152 received a mailed paper-based questionnaire with prepaid return envelope; one reminder was sent to non-responders. A total of 104 patients met the inclusion criteria and were included in the analysis (Fig. 1). This retrospective, monocentric, nonrandomized, open-label study was approved by the local Ethics Committee (166/2022BO1).

### Patient data

After informed consent was obtained, further perioperative data were retrieved from the patients’ files (Table 1 and 2). Based on preoperative histological findings and surgical complexity, patients were assigned to the following groups: those with benign histology and lesions with uncertain malignant potential were assigned to the outpatient group, while those with malignant diagnosis to the inpatient group.

**Table 1:** *Patient characteristics and perioperative data* (*US/Mx-guided WGL *  ultrasound/ mammography-guided wire-guided localization, *IOUS*   intraoperative ultrasound, *PE*   excisional biopsy, *BCT*   breast conserving therapy, *SNB*   sentinel-node-biopsy (‡ = t-test, † = Fisher exact test))

	Total mean ± SD (range) number | percentage	Outpatient group mean ± SD (range) number | percentage	Inpatient group mean ± SD (range) number | percentage	p-value
	n = 104	n = 57	n = 47	
Age [years]	60.0 ± 19.6 (20.8—92.4)	48.4 ± 15.8 (20.8—85.8)	74.1 ± 13.9 (40.9—92.4)	< 0.001‡
BMI [kg/m^2^]	24.6 ± 5.6 (15.8—45.3)	23.5 ± 5.5 (16.8—45.3)	26.0 ± 5.3 (15.8—40.9)	0.023‡
ASA-Score				< 0.001†
I	21 | 20.2%	18 | 31.6%	3|6.4%	
II	62|59.6%	39|68.4%	23|48.9%	
III	21|20.2%	0|0.0%	21|44.7%	
ECOG-Score				< 0.001†
0	85|81.7%	55|96.5%	30|63.8%	
1	16|15.4%	2|3.5%	14|29.8%	
2	1|1.0%	0|0.0%	1|2.1%	
3	2|1.9%	0|0.0%	2|4.3%	
Localization technique				0.820†
Wire-guided localization	59|56.7%	34|59.6%	25|53.2%	
US-guided WGL	48	25	23	
Mx-guided WGL	11	9	2	
Palpation guided/ IOUS	28|26.9%	17|29.8%	11|23.4%	
None	17|16.3%	6|10.5%	11|23.4%	
Type of Surgery				< 0.001†
PE/ Ductectomy	47|45.2%	46|80.7%	1|2.1%	
BCT	32|39.8%	11|19.3%	21|44.7%	
BCT + SNB	15|14.4%	0|0.0%	15|31.9%	
Mastectomy	3|2.9%	0|0.0%	3|6.4%	
Mastectomy + SNB	7|6.7%	0|0.0%	7|14.9%	
Duration of surgery [min]	39.5 ± 21.8 (10—108)	26.6 ± 11.7 (10—70)	55.1 ± 21.1 (16—108)	< 0.001‡
Specimen weight [g]	66.7 ± 170.0 (1—1103)	8.5 ± 12.2 (1—83)	137.3 ± 235.0 (5—1103)	< 0.001‡

**Table 2:** *Perioperative data (TLA and analgosedation) *(^‡^ = Wilcoxon-Mann–Whitney rank test, ^†^ = Fisher exact test)

	Total mean ± SD (range) number | percentage	Outpatient group mean ± SD (range) number | percentage	Inpatient group mean ± SD (range) number | percentage	p-value
	n = 104	n = 57	n = 47	
PREOPERATIVE				
Number of injections				
TLA 0.05%	61|58.7%	19|33.3%	42|89.4%	< 0.00†
TLA 0.21%	55|52.9%	38|66.7%	17|36.2%	0.003†
TLA-Volume [ml]				
Total	299.2 ± 210.1 (25.0 – 1000.0)	179.9 ± 127.5 (50.0 – 500.0)	443.9 ± 200.1 (25.0 – 1000.0)	< 0.00**‡**
Anaesthetic dose (amount of ropivacaine and lidocaine) [mg]				
	264.3 ± 119.7 (55.6 – 666.6)	223.2 ± 97.7 (58.8—511.1)	314.1 ± 125.9 (55.6 – 666.6)	< 0.00**‡**
INTRA-OPERATIVE				
Number of reinjections				
TLA 0.21%	49|47.1%	16|28.1%	33|70.2%	< 0.00†
TLA-Volume [ml]				
0.21%	15.9 ± 26.9 (0 – 160)	4.8 ± 9.9 (0 – 40)	29.3 ± 34.0 (0 – 160)	< 0.00‡
Anaesthetic dose 0.21% (amount of ropivacaine and lidocaine) [mg]				
	35.3 ± 59.7 (0.0 – 355.5)	10.6 ± 22.0 (0.0 – 88.8)	65.1 ± 75.6 (0.0 – 355.5)	< 0.00**‡**
TOTAL				
TLA-Volume [ml]	315.1 ± 221.4 (25 – 1000)	184.7 ± 131.4 (50 – 510)	473.3 ± 205.2 (25 – 1000)	< 0.00**‡**
Anaesthetic dose (amount of ropivacaine and lidocaine) [mg]				
	299.5 ± 143.1 (55.6 – 666.6)	233.8 ± 102.0 (81.0 – 577.7)	379.2 ± 146.0 (55.6 – 666.6)	< 0.00**‡**
Additional analgosedation				
Midazolam				
number	25|24.0%	6|10.5%	19|40.4%	< 0.00†
dose [mg]	1.5 ± 0.9 (0.5 – 3.5)	1.2 ± 0.6 (0.5—2.0)	1.6 ± 1.0 (0.5 – 3.5)	0.619**‡**
Piritramide				
number	5	0	5	0.012**‡**
dose [mg]	6.5 ± 1.6 (3.8 – 7.5)	± 0.0 (0.0 – 0.0)	6.5 ± 1.6 (3.8 – 7.5)	
Other	3	0	3	
No analgosedation	79|76.0%	51|89.5%	28|59.6%	

### Tumescent local anaesthesia (TLA)

**Fig. 2:**
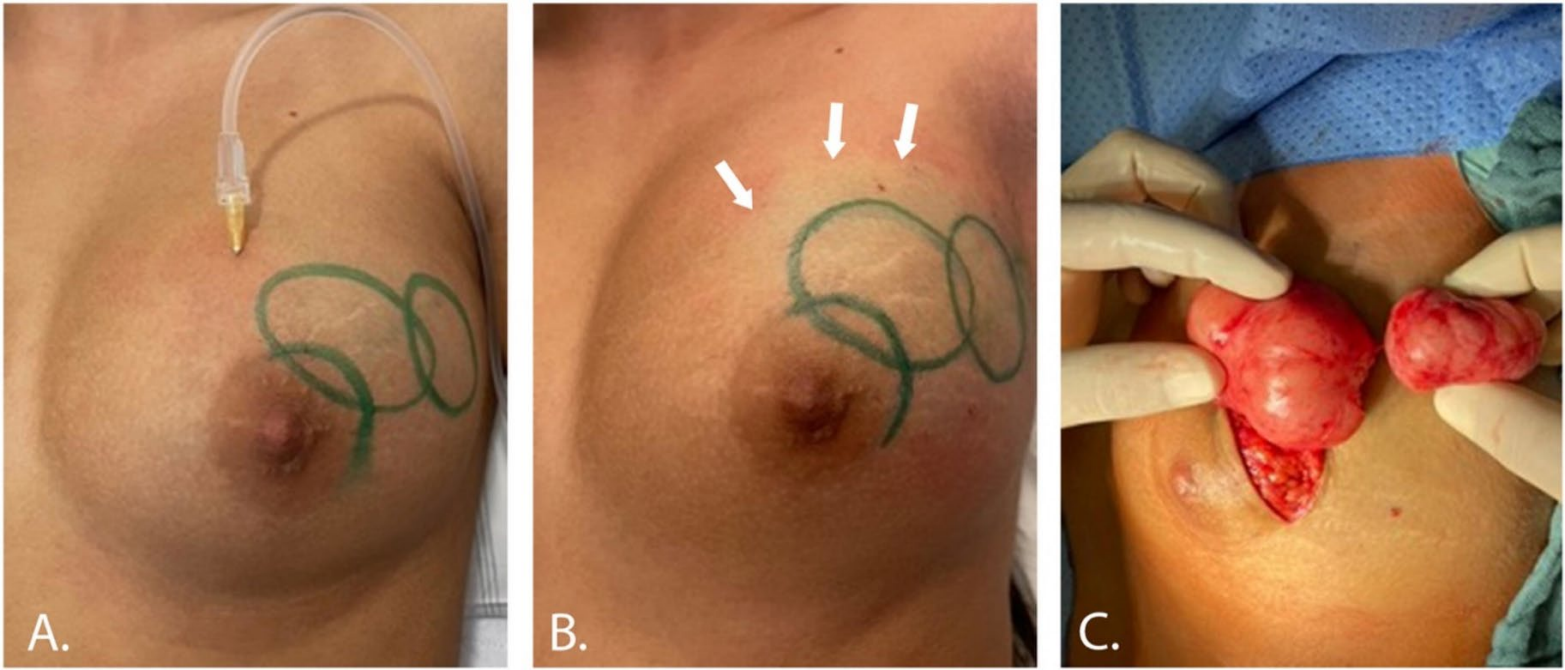
**A** Marking by the surgeon and start of TLA-infiltration **B**. Ready for surgery: typical anaemic aspect after TLA-infiltration (arrows) **C**. Demonstration of the fibroadenomas during surgery

Prior to surgery, the surgeon defined the infiltration area and the appropriate TLA concentration (0.05% or 0.21%), based on the surgical extent and duration of the surgery. 0.21% solution was mainly used for smaller interventions requiring limited infiltration volumes to achieve sufficient anaesthesia within a very short time. In contrast, 0.05% solution, with an onset time of approximately 20 min, was preferred in larger breasts and more extensive resections, where higher total infiltration volumes were necessary. The TLA solution consisted of a full electrolyte solution containing a combination of the short-acting local anaesthetic lidocaine and the long-acting local anaesthetic ropivacaine with the addition of epinephrine (Appendix 1). The infiltration was carried out at flow rates of 50–500 ml/h via infusion pump systems operated by trained personnel in a separate preparation room until the area exhibited the characteristic swollen and anaemic appearance indicative of effective anaesthesia (Fig. 2).

### Surgical procedure and additional intraoperative analgosedation

Surgery started 30 to 120 min after TLA-infiltration. Patients’ vital signs were monitored during TLA and surgery. Agitated patients were offered additional sedation with intravenous midazolam to reduce anxiety and stress. Further TLA solution (0.21%) was applied in the event of intraoperative pain, after localization of the exact position of the sentinel lymph node by gamma probe, or to place a drain. Otherwise, the surgical procedure did not differ from similar surgeries under GA. The surgeon’s satisfaction with the procedure was assessed postoperatively.

### Questionnaire

The questionnaire was developed at the Department of Women´s Health in Tuebingen and comprised a total of eight single-item questions (Appendix 2). Reasons for choosing surgery in TLA, intraoperative and postoperative pain, complications and general patient satisfaction were asked. Missing data were not imputed.

### Statistical methods

The statistical analysis was performed using SPSS^®^ (software version 29.0) and R (version 4.5.2.). Continuous data were summarized as mean, standard deviation (SD), minimum and maximum and differences between groups were tested using the t-test or Wilcoxon-Mann–Whitney rank test. For nominal data, numbers and proportions are calculated and the chi-square test or the Fisher exact test is used in case of weakly populated fields to test for differences. The relation between anaesthetic dose and body weight was assessed by the Pearson correlation coefficient (*r*). The significance level was set at 5%. Also, a multiple regression model was formulated for the total anaesthetic dose with factors BMI and extent of surgical procedures (5 levels). Quality of fit was assessed by coefficient of determination R^2^.

## Results

### Patient characteristics

The data of 104 included patients (103 female patients, 1 male patient) was analysed (Fig. 1). In the outpatient group, 24 patients had a benign histology confirmed by core needle biopsy and 33 a lesion with uncertain malignant potential or a suspicious finding such as BI-RADS 4 microcalcifications with a 3–94% probability of malignancy. The mean age of all patients was 60.0 years (SD 19.6 years), with significantly younger patients in the outpatient group (48.4 vs. 74.1 years; p < 0.001) (Table 1). The mean BMI of all patients was 24.6 kg/m^2^ (SD 5.6 kg/m^2^) with a significantly lower BMI of 23.5 kg/m^2^ (SD 5.5 kg/m^2^) in the outpatient group compared to the inpatient group (BMI 26.0 kg/m^2^ (SD 5.3 kg/m^2^); p = 0.023). Higher preoperative ASA and ECOG scores were significantly more common in the inpatient group (p < 0.001) (Table 1) with all 21 ASA III patients belonging to this group. 3.5% of outpatients had an ECOG score of 1, compared with 36.2% of inpatients with an ECOG score ≥ 1, reflecting the lower functional status in the latter group. Wire-guided localization (WGL) was used in 56.7% for preoperative localization (59.6% of outpatients; 53.2% of inpatients; Table 1). There were no reports of wire dislocation. Intraoperative ultrasound (IOUS)- or palpation-guided excision as the only localization technique was used in 28 patients (26.9%). Since group allocation was based almost exclusively on surgical complexity, the differences between the two groups regarding surgical complexity were statistically significant (p < 0.001). In total a simple probe excision was performed in 47 of 104 patients (45.2%). Breast-conserving therapy (BCT) was conducted in 32 patients (30.8%) with sentinel-node biopsy (SNB) in 15 patients (14.4%). Mastectomy was carried out in 10 patients (9.6%), of whom seven (6.7%) underwent additional SNB/ axillary exploration. The specimen weight was significantly higher in the inpatient group than in the outpatient group (137.3 ± 235.0 g vs. 8.5 ± 12.2 g, p < 0.001; Table 1).

### Tumescent local anaesthesia

In the outpatient setting, patients were administered more often 0.21% TLA solution preoperatively (38 vs. 17 patients) compared to inpatients with a mean volume of 71.9 ml (SD 64.5 ml) (Table 2). Within the inpatient group undergoing more complex procedures, the 0.05% solution was used more frequently preoperatively (42 vs. 19). Inpatients also received significantly higher infiltration volumes (473.3 mL (SD 205.0) vs. 184.7 mL (SD 131.4), p < 0.001, Appendix 3A) and a higher total dose of ropivacaine and lidocaine (379.2 mg (SD 146.0) vs. 233.8 mg (SD 102.0), p < 0.001, Appendix 3B).

The total anaesthetic dose per kilogram body weight was significantly higher in inpatients than in outpatients (median 5.5 mg vs. 3.7 mg; p < 0.001). However, the correlation between anaesthetic dose and body weight was weak in both groups (inpatients: *r* = 0.11; outpatients: *r* = 0.27;).

### Perioperative data and additional intraoperative analgosedation

Additional application of 0.21% TLA solution during surgery was necessary in 49 patients (47.1%), 16 belonging to the outpatient group (28.1%) with an average volume of 4.8 ml (SD 9.9 ml) and 33 to the inpatient group (70.2%) with an average volume of 29.3 ml (SD 34.0 ml) (p < 0.001; Table 2). 25 patients (24.0%) opted for discretional intraoperative sedation with Midazolam: 4/47 with excisional biopsies, 6/32 with BCT, 7/15 with BCT + SNB and 8/10 with mastectomy (with/without SNB). Midazolam was administered at an average dose of 1.5 mg (SD 0.9 mg) (outpatient group 1.3 mg (SD 0.6 mg) vs. inpatient group 1.6 mg (SD 1.0 mg)). The additional administration of piritramide (mean amount 6.5 mg, SD 1.6 mg) was necessary in the inpatient group in five cases. In three instances further analgesics (dexamethasone, metamizole or paracetamol) were necessary. There were no intraoperative complications, but the anaesthetist had to be consulted during one procedure in the inpatient group to optimize analgesia. In this case, 800 µg remifentanil i.v. was also administered via syringe infusion pump. The average duration of surgery was 39.5 min (SD 21.8 min), with significantly shorter procedures in the outpatient group (26.6 ± 11.7 min vs. 55.1 ± 21.1 min, p < 0.001; Table 1). The surgeon’s satisfaction was documented in 96.2%. The five surgeons awarded high satisfaction scores of 8 to 10 (on a scale of 0 to 10) for 93 surgical procedures (93.0%), of which 55 (98.2%) were attributable to the outpatient group and 38 (86.3%) to the inpatient group (p = 0.039). Seven surgeries were scored 7 (three times in case of axillary surgery) and one mastectomy scored 5, mostly due to insufficient pain control. Postoperatively, 6 out of 56 patients (10.7%) of the outpatient group received a malignant diagnosis.

### Regression model for total anaesthetic dose

A regression model for total anaesthetic dose was formulated with factors BMI and surgery type (with levels 1 to 5 representing surgical complexity). The coefficient of determination R^2^ was 0.47 and BMI as well as surgery type were significant (p = 0.004 respectively p < 0.001).

### Questionnaire

The time difference between surgery and answering the questionnaire was on average 5.4 months (SD 3.0 months, min 1.1, max 13.0 months) and was similar in both groups (5.3 months vs. 5.4 months; p = 0.881). The first question asked about patient satisfaction with surgery with five response options. All inpatients and altogether 96.1% answered this question with very satisfied or satisfied (p = 0.022, Table 3). The perception of local anaesthesia application before surgery was assessed using a five-point scale (p = 0.469, Table 3). 86 patients (82.7%) perceived the TLA as more pleasant than expected or neutral, of which 48 were assigned to the outpatient group (84.2%) and 38 to the inpatient group (80.9%). Only three outpatients (5.3%) and two inpatients (4.3%) reported pain during the TLA-application. 87.5% were satisfied or very satisfied with the organization around the surgery (p = 0.273, Table 3). 94 of 104 patients (90.4%) expressed willingness to undergo the surgery again under TLA, with 95.7% of patients in the inpatient group indicating the same (p = 0.140). Additionally, 93 patients (89.4%) would recommend similar surgery using TLA to a friend (p = 0.207, Table 3).

**Table 3:** *Excerpt from the questionnaire (***‡** = Wilcoxon-Mann–Whitney rank test, † = Fisher exact test) (complete questionnaire see Appendix 2)

Questionnaire	Total number | percentage	Outpatient group number | percentage	Inpatient group number | percentage	p-value
	n = 104	n = 57	n = 47	
Satisfaction with the surgery				
Very satisfied	77|74.0%	37|64.9%	40|85.1%	0.022‡
Satisfied	23|22.1%	16|28.1%	7|14.9%	
Neutral	2|1.9%	2|3.5%	0|0.0%	
Dissatisfied	1|1.0%	1|1.8%	0|0.0%	
Very dissatisfied	0|0.0%	0|0.0%	0|0.0%	
No answer	1|1.0%	1|1.8%	0|0.0%	
Surgery performed again in local anaesthesia retrospectively				
Yes	94|90.4%	49|86.0%	45|95.7%	0.176†
No	9|8.7%	7|12.3%	2|4.3%	
No answer	1|1.0%	1|1.8%	0|0.0%	
Recommendation to a friend				
Yes	93|89.4%	49|86.0%	44|93.6%	0.337†
No	11|10.6%	8|14.0%	3|6.4%	
Sensation of local anaesthesia				
More pleasant than expected	44|42.3%	26|45.6%	18|38.3%	0.472‡
Neutral/ as expected	42|40.4%	22|38.6%	20| 42.6%	
More unpleasant than expected	13|12.5%	6|10.5%	7|14.9%	
Painful	5|4.8%	3|5.3%	2|4.3%	
Very painful	0|0.0%	0|0.0%	0|0.0%	
Satisfaction with organization				
Very satisfied	58|55.8%	30|52.6%	28|59.6%	0.275‡
Satisfied	33|31.7%	17|29.8%	16|34.0%	
Neutral	6|5.8%	4|7.0%	2|4.3%	
Dissatisfied	4|3.8%	4|7.0%	0|0.0%	
Very dissatisfied	3|2.9%	2|3.5%	1|2.1%	

51.0% of patients reported no postoperative pain. Postoperative pain was significantly more frequent in outpatients than in inpatients (64.9% vs. 29.8%, p < 0.001). 21.2% reported pain not requiring analgesics, 23.1% used pain medication for up to three days. Prolonged analgesic use was observed in five outpatients (8.8%). Despite undergoing more extensive procedures, inpatients required analgesics significantly less often and for a shorter duration than outpatients (29.8% vs. 64.9%, p < 0.001). Reasons for choosing TLA were assessed via a multiple-choice questionnaire (Appendix 4). Most patients (61.2%) chose TLA based on surgeon recommendation. Organizational advantages were cited by 61.4% of outpatients, while 52.2% of inpatients preferred TLA due to comorbidities or advanced age. Additional factors included avoidance of physical stress from GA, prior GA experiences, and better overall management. Postoperative complications were assessed by patient self-report using the questionnaire and verified through review of the medical records. 94.2% had no complications (98/104). Six patients (5.8%) had postoperative complications grade 2 or 3 according to Clavien-Dindo [9] (Table 4): one inpatient required surgery for postoperative bleeding, one outpatient for a breast abscess, and four patients received oral antibiotics for wound infection.

**Table 4:** *Postoperative complications by Clavien-Dindo *[9]

		Total number | percentage	Outpatient group number | percentage	Inpatient group number | percentage
		n = 104	n = 57	n = 47
	Complications			
	No	98 | 94.2%	54 | 94.7%	44 | 93.6%
	Yes	6 | 5.8%	3 | 5.3%	3 | 6.4%
Clavien-Dindo grade 2	Wound infection	4 | 3.8%	2 | 3.5%	2 | 4.3%
Clavien-Dindo grade 3	Postoperative bleeding	1 | 1.0%	0 | 0.0%	1 | 2.1%
	Abscess	1 | 1.0%	1 | 1.8%	0 | 0.0%

## Discussion

### Patient characteristics

Breast cancer is the most frequent cancer in women, and its incidence is increasing in the elderly [10–12]. In our study, older patients (74.1 vs. 48.4 years; *p* < 0.001) with higher preoperative ASA and ECOG scores appeared significantly more often with a malignant diagnosis in the inpatient group (*p* < 0.001). TLA is a convenient alternative to GA in a broad spectrum of breast surgeries for most patients, but especially for elderly or high-risk patients (ASA > II and ECOG ≥ 1). Despite the potential need for supplementary short-acting sedation, TLA facilitates postoperative mobilisation and recovery [13] without the typical post-anaesthetic downtime and avoids the possible intensive care after GA [14]. TLA is also an appealing option for minimally invasive diagnostic procedures to clarify suspicious lesions that cannot be biopsied, as further oncological surgeries may be necessary. In this study, 10.7% (6/56) were diagnosed with malignancy postoperatively, necessitating further surgical intervention.

### Tumescent local anaesthesia

This study aimed to estimate typical TLA volumes required for adequate breast anaesthesia to support the broader adoption of this method. Outpatients with minor procedures received 0.21% TLA at an average of 184.7 ml, whereas inpatients received 0.05% TLA with at an average of 473.3 ml as the higher volume allows larger breast areas to be infiltrated. The total anaesthetic dose per kilogram body weight was significantly higher in inpatients than in outpatients (*p* < 0.001). However, the correlation between anaesthetic dose and body weight was only weak. Surgical complexity as well as duration of surgery appear to be more relevant than body weight.

Despite a swollen, wet surgical site, surgeon satisfaction was high, with no wire dislocations and facilitated tissue preparation due to hydrodissection. Due to the addition of epinephrine, the surgeon also benefits from an anaemic operating field [15, 16]. Surgeon satisfaction was significantly higher for outpatient procedures compared to inpatient procedures (98.2% versus 86.3%, *p *= 0.039), likely due to fewer intraoperative re-injections (16 vs. 33, *p* < 0.001) and shorter surgery duration (26.6. vs. 55.1 min, *p* < 0.001). The preoperative TLA infusion caused discomfort in five patients (4.8%), highlighting the importance of adjusting the infiltration rate, infusion speed and cannula position individually [17] [18].

The oncological implications of TLA in breast cancer surgery warrant consideration in the context of current evidence. Badwe et al. [19] demonstrated that peritumoral infiltration of lidocaine prior to surgery significantly improves overall and disease-free survival in breast cancer patients, suggesting a protective effect against surgery-induced tumour dissemination. Since TLA solution contains lidocaine as a key component, it is highly conceivable that TLA may offer similar oncological benefits, beyond its primary anaesthetic efficacy.

### Perioperative data and additional intraoperative analgosedation

During surgery, 0.21% TLA was used significantly more often and in higher volumes in the inpatient group, particularly for drain placement, SN localization, and due to more complex surgical procedures. 25 patients (24.0%) requested additional sedation (1.5 ± 0.9 mg midazolam), more frequently among inpatients (24% vs. 10.5%), likely reflecting higher anxiety levels and longer surgical procedures. Midazolam was rarely required for procedures with limited surgical extent (PE/BCT) but was administered more frequently in more extensive surgeries, such as BCT + SNB or mastectomy with or without SNB. Schnabl et al. reported additional sedation with midazolam 3.6 mg in 42.0% of geriatric patients with head and neck skin tumours as a safe and effective approach [8].

Inpatient surgeries were significantly longer than outpatient procedures (55.1 min (SD 21.1 min) vs 26.6 min (SD 11.7 min), *p* < 0.001), reflecting the type and invasiveness of surgery. In the literature, breast cancer patients with ASA score III or IV underwent total mastectomy in an average of 35–55 min [6, 20]. As this study includes a wide range of surgical procedures, a comparison of surgical times is not feasible. TLA seemed to have no negative effect on the duration of the surgery. TLA facilitates organizational workflows [13] since it can be safely administered in various clinical settings without requiring an anaesthetist.

### Postoperative analgesics

In the prospective study by Eckhardt, 32.0% of patients reported pain 48 h after SNB in TLA, with 17.3% requiring additional analgesics [21]. In the present study postoperative pain management was not mandated by a strict study protocol but was administered on an individual basis. 51 patients (49.0%) recalled postoperative pain, with 29 requiring analgesics. Inpatients needed significantly less and shorter courses of analgesics than outpatients (29.8% vs. 64.9%, *p* < 0.001), likely due to higher preoperative TLA doses (379.2 mg vs. 233.8 mg, *p* < 0.001), more intraoperative re-injections (33 vs. 16, *p* < 0.001), and standardized inpatient pain regimens. Ropivacaine’s long-acting effect may have contributed to sustained postoperative analgesia [22, 23]. However, the relative contribution of each factor remains uncertain and warrants further investigation in prospective studies.

### Complications

The literature on postoperative wound infections is imprecise, but meta-analyses suggest risk factors such as advanced age, comorbidities, ASA ≥ III, high BMI and postmenopausal status [24]. Eckhardt et al. reported wound infections requiring antibiotics in 3.3% of elderly patients undergoing surgery in TLA [21]. In our study, the rate was similar (4.8%), including one abscess requiring surgical intervention, likely attributable to the same risk factors.

### Patient satisfaction (questionnaire)

Data on patient satisfaction with breast surgery under TLA are scarce. Eckhardt et al. reported 96.7% satisfaction and 97.3% recommendation rates after SNB in 150 patients [21]. In line with these results, 96.1% of our patients were satisfied, with all 47 inpatients reporting a very high level of satisfaction (100.0%), despite more extensive procedures. Overall, 90.4% would choose TLA again, and 89.4% would recommend it to others.

### Limitations

Several methodological limitations of this study must be acknowledged. First, the primary endpoint was assessed using a non-validated, locally developed questionnaire, as existing standard measures do not capture the specific clinical nuances of breast surgery under TLA. This lack of formal validation, combined with a potential non-response bias from missing follow-up reminders, may limit the generalizability and comparability of our findings. Second, the retrospective design with a mean interval of 5.4 months between surgery and survey completion may have introduced recall bias. Third, the absence of a GA control group precludes direct comparative conclusions, and potential confounding factors exist due to the varying experience of the five operating surgeons.

## Conclusion

To our knowledge, this is the first study demonstrating that TLA is a feasible and effective alternative to GA for breast surgery in a routine clinical setting, particularly in elderly patients. Mean infiltration volumes of 185 ± 130ml (0.21%) are sufficient for minor procedures, whereas larger excisions require 475 ± 200ml (0.05%). Hydrodissection facilitates tissue preparation and goes along with high satisfaction of the surgeons. High patient satisfaction and recommendation rates, combined with a low incidence of complications, support TLA as a viable alternative for breast surgery.

Future prospective studies with larger sample sizes, validated standardized PROMs, GA control groups, and comprehensive assessment of anatomical and procedural outcomes are needed to enable robust comparisons and further define the oncological and clinical role of TLA within the expanding framework of anaesthesia techniques in senology.

## Supplementary Information

Below is the link to the electronic supplementary material.

Supplementary file1 (PDF 24 KB)

Supplementary file2 (PDF 128 KB)

Supplementary file3 (PDF 348 KB)

Supplementary file4 (PDF 29 KB)

## Data Availability

No datasets were generated or analysed during the current study.
