# Supplementary material for: Patient satisfaction and perioperative data after breast surgery in tumescent local anaesthesia
Source: Arch Gynecol Obstet. 2026 Jul 18;313(1):230. doi: 10.1007/s00404-026-08524-x (PMC13380592; doi:10.1007/s00404-026-08524-x)
Supplement: Supplementary file 1 — Supplementary file1 (PDF 24 KB) [file 404_2026_8524_MOESM1_ESM.pdf]

Appendix 1. TLA-solutions with *stock solution = ropivacaine 10mg (1%) + lidocaine 20mg (2%) per 1ml)

	**TLA 0.21%**	**TLA 0.05%**
Isotonic complete electrolyte solution (Jonosteril®)	500ml	
Epinephrine (1: 1 000 000)	0.5ml	
Stock solution*	50ml	20ml
recommended maximum dose	3ml per kg body weight	12ml per kg body weight
Start of effect (min)	2-5	20-30
