# Supplementary material for: Patient satisfaction and perioperative data after breast surgery in tumescent local anaesthesia
Source: Arch Gynecol Obstet. 2026 Jul 18;313(1):230. doi: 10.1007/s00404-026-08524-x (PMC13380592; doi:10.1007/s00404-026-08524-x)
Supplement: Supplementary file 2 — Supplementary file2 (PDF 128 KB) [file 404_2026_8524_MOESM2_ESM.pdf]

Universitätsklinikum Tübingen

Verantwortliche Ärztin
Dr. Bettina Böer
Department für Frauengesundheit
Calwerstr. 7
72076 Tübingen
Tel: 07071-29 82211
Email: Bettina.boeer@med.uni-tuebingen.de

Universitätsklinikum Tübingen

Department für Frauengesundheit

Geschäftsführende Ärztliche Direktorin
Prof. Dr. med. Sara Brucker

Universitäts-Frauenklinik
Ärztlicher Direktor
Prof. Dr. med. Dr. h. c. mult. Diethelm Wallwiener
FRCOG

Forschungsinstitut für Frauengesundheit

Ärztliche Direktorin
Prof. Dr. med. Sara Brucker

FRAGEBOGEN: "Retrospektive Datenerhebung bei Patientinnen nach senologischen Operationen in Tumeszenz-Lokalanästhesie"

Datum: ________________ Studien-ID: ________________

Frage 1:
Wie zufrieden waren Sie insgesamt mit der Operation?

- ☐ sehr zufrieden
- ☐ zufrieden
- ☐ neutral
- ☐ unzufrieden
- ☐ sehr unzufrieden

Frage 2:
Würden Sie die Operation rückblickend wieder in Lokalanästhesie durchführen lassen?

- ☐ Ja
- ☐ Nein

Frage 3:
Würden Sie einer Freundin eine ähnliche Operation in Lokalanästhesie empfehlen?

- ☐ Ja
- ☐ Nein

Frage 4:
Warum haben Sie sich für eine Operation in Lokalanästhesie entschieden?
(auch Mehrfachnennungen möglich)

- ☐ Ich habe gute Erfahrungen mit Operationen in Lokalanästhesie gemacht.
- ☐ Ich habe schlechte Erfahrungen mit Operationen in Vollnarkose gemacht.
- ☐ Das wurde mir von meinem/r Arzt/ Ärztin empfohlen.
- ☐ Aufgrund meiner Vorerkrankungen/ Alters wollte ich keine Vollnarkose.
- ☐ Ich wollte die Tumorentfernung/ Operation miterleben.
- ☐ Ich schätze die organisatorischen Vorteile der Lokalanästhesie (man darf essen/ trinken, man kann früher entlassen werden, etc).
- ☐ Persönliche Gründe: ______________________________

Frage 5:
Wie haben Sie die lokale Betäubung vor der Operation empfunden?

- ☐ angenehmer als erwartet
- ☐ neutral/ wie erwartet
- ☐ unangenehmer als erwartet
- ☐ schmerzhaft
- ☐ sehr schmerzhaft

Frage 6:
Wie zufrieden waren Sie mit der Organisation rund um die Operation?

- ☐ sehr zufrieden
- ☐ zufrieden
- ☐ neutral
- ☐ unzufrieden
- ☐ sehr unzufrieden

Frage 7:
Hatten Sie nach der Operation Schmerzen im Operationsgebiet?

- ☐ Nein
- ☐ Ja, ich benötigte aber keine Schmerzmittel
- ☐ Ja, ich nahm deshalb Schmerzmittel für maximal drei Tage nach der Operation.
- ☐ Ja, ich nahm deshalb Schmerzmittel für mehr als drei Tage nach der Operation.

Frage 8:
Traten nach der Operation eine oder mehrere Komplikationen auf?

- ☐ keine Komplikationen
- ☐ Nachblutung mit zusätzlicher Operation
- ☐ Antibiotika-Einnahme wegen Wundinfektion
- ☐ andere: ______________________________

Universitätsklinikum
Tübingen

Responsible Physician
Dr. Bettina Böer
Department of Women's Health
Calwerstr. 7
72076 Tübingen, Germany
Tel: 07071-29 82211
Email: Bettina.boeer@med.uni-tuebingen.de

QUESTIONNAIRE: "Retrospective data collection in patients after breast surgery in Tumescent Local Anaesthesia"

Date: _______________ Study-ID: _______________

Question 1:
How satisfied were you overall with the surgery?

- ☐ very satisfied
- ☐ satisfied
- ☐ neutral
- ☐ dissatisfied
- ☐ very dissatisfied

Question 2:
In retrospect, would you undergo the surgery again under local anaesthesia?

- ☐ yes
- ☐ no

Question 3:
Würden Sie einer Freundin eine ähnliche Operation in Lokalanästhesie empfehlen?

- ☐ Ja
- ☐ Nein

Question 4:

Why did you choose to have the surgery under local anaesthesia? (Multiple answers possible)

- ☐ I have had positive experiences with surgery under local anaesthesia.
- ☐ I have had negative experiences with surgery under general anaesthesia.
- ☐ It was recommended to me by my physician.
- ☐ Due to my comorbidities/age, I wanted to avoid general anaesthesia.
- ☐ I wanted to be awake during the tumour removal/surgery.
- ☐ I appreciate the organisational advantages of local anaesthesia (being allowed to eat/drink, earlier discharge, etc.).
- ☐ Personal reasons: ______________________________________

Question 5:

How did you perceive the local anaesthesia before surgery?

- ☐ More pleasant than expected
- ☐ neutral/ as expected
- ☐ More unpleasant than expected
- ☐ painful
- ☐ very painful

Question 6:

How satisfied were you with the organisation surrounding the surgery?

- ☐ very satisfied
- ☐ satisfied
- ☐ neutral
- ☐ dissatisfied
- ☐ very dissatisfied

Question 7:

Did you experience pain in the surgical area after the operation?

- ☐ no
- ☐ Yes, but I did not require analgesics
- ☐ Yes, I took analgesics for up to three days after surgery
- ☐ Yes, I took analgesics for more than three days after surgery

Question 8:

Did any complications occur after the surgery?

- ☐ no complications
- ☐ postoperative bleeding requiring additional surgery
- ☐ Antibiotic treatment due to wound infection
- ☐ other: ______________________________________
