# Supplementary material for: Patient satisfaction and perioperative data after breast surgery in tumescent local anaesthesia
Source: Arch Gynecol Obstet. 2026 Jul 18;313(1):230. doi: 10.1007/s00404-026-08524-x (PMC13380592; doi:10.1007/s00404-026-08524-x)

Appendix 3.

A. Box plots of TLA-volumes in both groups

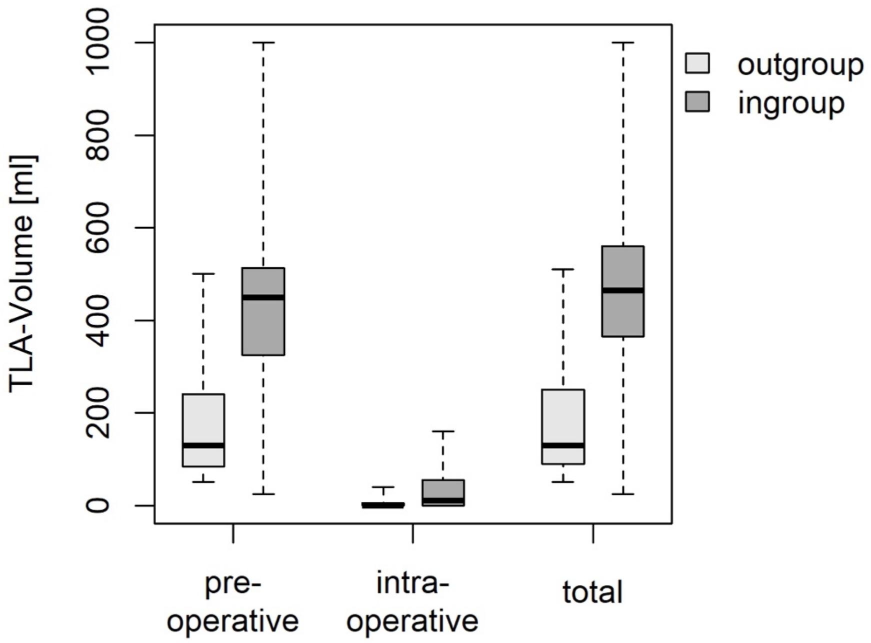

B. Box plots of amount of ropivacaine and lidocaine in both groups

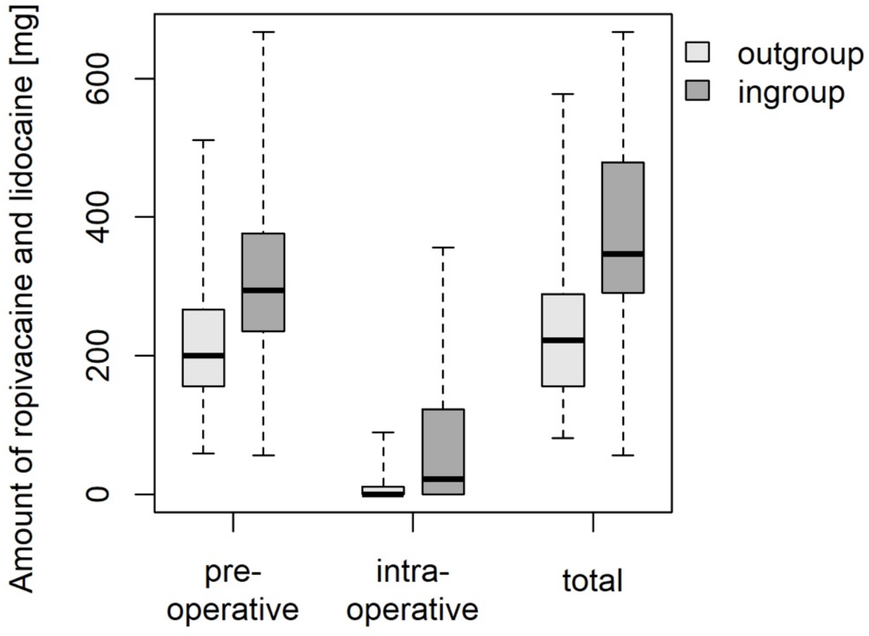

Supplement: Supplementary file 3 — Supplementary file3 (PDF 348 KB) [file 404_2026_8524_MOESM3_ESM.pdf]
