# Supplementary material for: Patient satisfaction and perioperative data after breast surgery in tumescent local anaesthesia
Source: Arch Gynecol Obstet. 2026 Jul 18;313(1):230. doi: 10.1007/s00404-026-08524-x (PMC13380592; doi:10.1007/s00404-026-08524-x)
Supplement: Supplementary file 4 — Supplementary file4 (PDF 29 KB) [file 404_2026_8524_MOESM4_ESM.pdf]

Appendix 4: Reasons for choosing TLA

**Reasons for choosing TLA**<br>(multiple answers possible)	**Total**<br>number | percentage<br>**n=103**	**Outpatient group**<br>number | percentage<br>**n=57**	**Inpatient group**<br>number | percentage<br>**n=46**
Good experiences with local anaesthesia	11 | 10.7%	7 | 12.3%	4 | 8.7%
Bad experiences with general anaesthesia	11 | 10.7%	3 | 5.3%	8 | 17.4%
Recommended by a medical doctor	63 | 61.2%	39 | 68.4%	24 | 52.2%
Pre-existing illnesses/ age	29 | 28.2%	5 | 8.8%	24 | 52.2%
Witnessing tumour removal/ surgery	13 | 12.6%	6 | 10.5%	7 | 15.2%
Appreciating the benefits	54 | 52.4%	35 | 61.4%	19 | 41.3%
Personal reasons	30 | 29.1%	18 | 31.6%	12 | 26.1%
